# 3C-Silicon Carbide Microresonators for Timing and Frequency Reference

**DOI:** 10.3390/mi7110208

**Published:** 2016-11-15

**Authors:** Graham S. Wood, Boris Sviličić, Enrico Mastropaolo, Rebecca Cheung

**Affiliations:** 1 Scottish Microelectronics Centre, Institute for Integrated Micro and Nano Systems, School of Engineering, University of Edinburgh, Edinburgh EH9 3FF, UK; e.mastropaolo@ed.ac.uk (E.M.); r.cheung@ed.ac.uk (R.C.); 2 Department of Marine Electronics and Communications, Faculty of Maritime Studies Rijeka, University of Rijeka, Rijeka HR-51000, Croatia; svilicic@pfri.hr

**Keywords:** silicon carbide, resonators, actuation methods, frequency tuning

## Abstract

In the drive to miniaturise and integrate reference oscillator components, microelectromechanical systems (MEMS) resonators are excellent candidates to replace quartz crystals. Silicon is the most utilised resonator structural material due to its associated well-established fabrication processes. However, when operation in harsh environments is required, cubic silicon carbide (3C-SiC) is an excellent candidate for use as a structural material, due to its robustness, chemical inertness and high temperature stability. In order to actuate 3C-SiC resonators, electrostatic, electrothermal and piezoelectric methods have been explored. Both electrothermal and piezoelectric actuation can be accomplished with simpler fabrication and lower driving voltages, down to 0.5 V, compared to electrostatic actuation. The vibration amplitude at resonance can be maximised by optimising the design and location of the electrodes. Electrical read out of the resonator can be performed with electrostatic or piezoelectric transduction. Finally, a great deal of research has focused on tuning the resonant frequency of a 3C-SiC resonator by adjusting the DC bias applied to the electrodes, with a higher (up to 160-times) tuning range for electrothermal tuning compared to piezoelectric tuning. Electrothermal tuning lowers the frequency, while piezoelectric tuning can be used to raise the frequency.

## 1. Introduction

Since the early 1940s, electromechanical oscillators have been used as the main components for time and frequency reference in consumer and commercial electronic products. Nowadays, reference oscillators represent a multi-billion dollar market. For implementing reference oscillators, quartz crystals offer advantages, such as high quality (*Q*) factors, low phase noise, excellent reliability and frequency stability over time and temperature and, therefore, are usually preferred over other bulk piezoelectric devices. The need for miniaturisation and integration into portable electronics has led manufacturers to push for shrinking the size of quartz oscillators (about 1.5 mm${}^{3}$). However, quartz crystals are still relatively bulky compared to typical components used in integrated circuits (IC). In addition, fabrication and encapsulation require the quartz crystal to be packaged separately from IC components.

In the last decade, the strong market push for optimising the performance and reducing the cost of smart devices has led research and development (R&D) companies to try to replace quartz oscillators with micro-fabricated silicon-based oscillators. There have been a considerable number of research groups and start-up companies that have invested great effort into the development of oscillators based on microelectromechanical systems (MEMS) as a technology for replacing their quartz counterparts [1]. In particular, much attention has been focused on the design and fabrication of microelectromechanical resonators as the fundamental building block of the overall oscillator [2]. Furthermore, MEMS resonators are also very attractive for performing filtering with a wide tunable range and thus for replacing filter banks in multiband communication systems and wide-band tracking receivers [3,4,5]. MEMS resonators that operate in a variety of resonant modes have been manufactured, and their operation has been demonstrated from the low frequency to ultra high frequency range with very low energy losses (i.e., high *Q*-factors).

Most common materials used for manufacturing MEMS resonators include silicon (Si), nitrides and oxides, with Si being the preferred one by companies that commercialised very successful devices (SiTIME and Integrated Device Technology [6]). At the present time, the integration of MEMS resonators on an IC follows two main approaches: hybrid integration (MEMS chip fabricated separately from IC chip and then packaged together) and direct fabrication (the MEMS device is fabricated directly on the IC chip). A wide variety of structural design and transduction mechanisms (e.g., electrostatic, electrothermal, piezoelectric and magnetomotive) has been investigated in the literature and has been well summarised in other extensive review papers [1,2,7,8].

An excellent alternative to Si is represented by silicon carbide (SiC), especially for applications requiring operation at temperatures above 500 °C and/or chemical inertness. With outstanding mechanical strength, high sublimation temperature and extreme inertness to most chemicals, SiC is well capable to withstand harsh environments and thus represents an obvious candidate to substitute Si, especially when highly reliable devices are required [9,10,11,12]. SiC’s electrical and mechanical properties have been shown to be highly stable at temperatures up to 600 °C [11,13]. A comprehensive review with historical perspective and developments on the growth and machining of single crystalline and polycrystalline SiC is presented in [14], and different polytypes of SiC can be used including hexagonal (4H-SiC, 6H-SiC) and cubic (3C-SiC). 3C-SiC has been shown to be particularly suitable for growth on Si substrates and surface micromachining, and therefore, processing of 3C-SiC can simplify the manufacturing process of MEMS structures having one or more degrees of freedom [15]. A variety of work has reported on 3C-SiC resonators favouring mainly surface micromachined vertical and lateral vibrating structures. MEMS resonators can benefit from SiC’s high acoustic velocity (and high Young’s modulus to mass density ratio), thus achieving higher operating frequencies compared to Si devices [12]. There are different examples of the remarkable performance of SiC resonators, which are reported in [16,17,18,19].

Most of the MEMS resonators presented in the literature utilise electrostatic transduction for actuation, read out and eventually for tuning the resonant frequency. However, disadvantages affecting electrostatically-actuated devices include relatively complex fabrication solutions to define the sub-micrometric gaps between the moving structures and the fixed actuation/sensing electrodes. In addition, electrostatically-transduced devices often exhibit relatively high motional impedances and large operating voltages. Therefore, more recent research has been directed towards electrothermal and piezoelectric transduction mechanisms. Furthermore, electrothermal transduction has been demonstrated to be a viable solution for performing electromechanical mixing and filtering [20]. SiC is particularly suitable for electrothermal excitation due to its high thermal conductivity facilitating the response of device temperature to Joule heat (in fact, actuation efficiency is maximised when the device temperature follows the drive voltage instantaneously) [21].

The stability of resonant frequency at different operating conditions is crucial for the successful employment of MEMS resonators as the time and frequency reference. Although the frequency stability of poly-Si and SiC resonators is comparable to quartz crystals nowadays, the shift of operating frequency due to fabrication variability and operating conditions still represents one of the main issues to be solved. In fact, the resonant frequency of MEMS devices cannot be fine-tuned as quartz crystals (i.e., laser cut after manufacturing to adjust the frequency accurately [22]). Therefore, tuning of resonant frequency is of utmost importance in order to compensate frequency shifts due to fabrication variability and thermal drift and, furthermore, if one were to use MEMS resonators for mechanical signal processing where frequency tracking is required [23].

This paper has the aim of reviewing the most relevant literature in the field of 3C-SiC MEMS resonators. Particular attention is focused on the different transduction methods used for actuation and sensing (i.e., read out) and on the design of the resonating structure together with the configuration of input and output ports. Furthermore, the paper discusses the strategies for tuning the frequency of the structures and highlights the degree of frequency shifts achieved by the different transduction methods.

The paper is divided into the following sections: Section 2 introduces the theory behind the transduction mechanisms that can be used on 3C-SiC resonators; Section 3 reviews the structures’ design and common fabrication processes for making 3C-SiC suspended structures; Section 4 and Section 5 summarise work and major results presented in the literature, divided for actuation methods (for driving the structures) and for sensing methods (for reading out); Section 6 focuses on the different tuning techniques used for shifting the devices’ resonant frequency; Section 7 completes the paper with conclusions and further remarks.

## 2. Transduction Mechanisms for SiC Resonators

The ability of converting input energy into a mechanical displacement, and vice versa, is of fundamental importance in MEMS structures. Transduction on MEMS can be performed using electrostatic, electrothermal, piezoelectric, piezoresistive, optical and magnetic techniques for actuation and/or sensing. This paper focuses mainly on transduction techniques, which enable the actuation of the structures (fundamental requirement to utilise them as the time and frequency reference). For instance, piezoresistive transduction can be used only for sensing in SiC structures, and in the literature, only the detection of static deflections has been reported (i.e., in pressure sensors [24]). To the authors’ knowledge, there are no works reporting piezoresistive sensing of vibration in SiC resonators. In addition, the implementation of piezoresistive sensing typically utilises a Wheatstone bridge configuration that requires an external bias voltage (thus increasing design and circuitry complexity). On the other hand, piezoelectric transduction can be used for both actuation and sensing and does not require an external bias voltage. The following sections summarise the theory behind electrostatic, electrothermal and piezoelectric transduction methods, which are used widely as reported in the literature for actuating and/or sensing on SiC MEMS resonators.

### 2.1. Electrostatic Actuation

**Figure 1:**
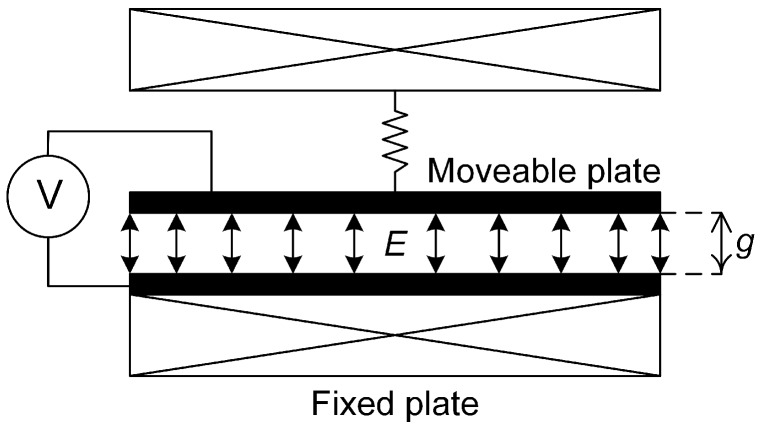
Mass-spring model representation of electrostatic transduction on a microelectromechanical systems (MEMS) resonator.

An MEMS resonator can be actuated electrostatically by positioning a fixed electrode parallel to the moveable structure, thus forming a two-plate capacitor, as illustrated in Figure 1. If two different voltages are applied to the electrode and the movable plate, then the potential difference created will result in an attractive force between them. The force, *F*${}_{\mathrm{elec}}$, acting between the plates as a result of a potential difference, *V*, is given by:

(1) $$F_{\mathrm{elec}}=\frac{\varepsilon AV^{2}}{2g^{2}}$$

where *A* is the overlapping area between the resonator and electrode plates, *g* is the gap separating them and ε is the permittivity [25]. It can be seen from Equation (1) that the force, *F*${}_{\mathrm{elec}}$, is proportional to the square of the voltage, *V*.

If the applied voltage signal, *V*, has both an alternating current (AC) and a direct current (DC) component, then *V*${}^{2}$ is given by:

(2) $$\begin{array}{cc} V^{2} & ={\left(V_{\mathrm{ac}}sin\omega t+V_{\mathrm{dc}}\right)}^{2}\\ & =2V_{\mathrm{ac}}V_{\mathrm{dc}}sin\omega t+0.5{V_{\mathrm{ac}}}^{2}\left(1-cos2\omega t\right)+{V_{\mathrm{dc}}}^{2} \end{array}$$

where ω is the angular frequency of *V*${}_{\mathrm{ac}}$.

From Equation (2), it can be seen that the first term will drive the structure into resonance if the frequency of the applied AC voltage is equal to the resonant frequency, *f*${}_{0}$, so that ω = 2π*f*${}_{0}$, and that the second term will drive the structure into resonance when the AC voltage has a frequency, such that ω = π*f*${}_{0}$.

### 2.2. Electrothermal Actuation

**Figure 2:**
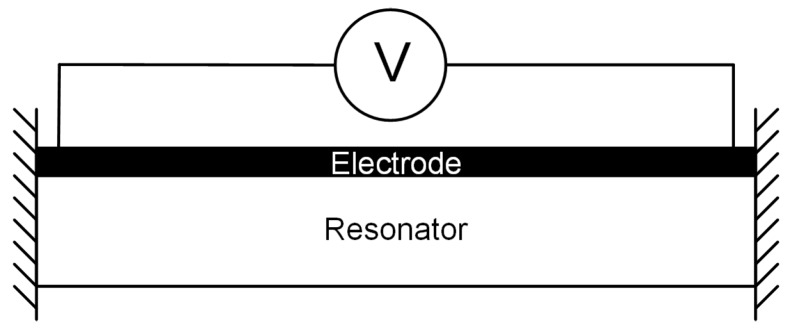
Schematic of electrothermal transduction on an MEMS resonator.

Electrothermal actuation of an MEMS resonator is achieved by inducing a thermal expansion, with advantages including relatively low operating voltages and simplified fabrication [26]. Usually, the structure that is to be actuated has an electrode material positioned on top, creating a bimaterial structure, as shown in Figure 2. When a voltage, *V*, is applied across the electrode, electric current is dissipated, and Joule heat is generated as a result of the electrode resistance. A temperature gradient, Δ*T*, is created within the structure, which leads to a mechanical strain, which is increased as a consequence of the difference between the thermal coefficients of expansion (TCE) of the two materials. The force created by the Joule heating is proportional to the power dissipated in the electrode, which is given by:

(3) $$P=\frac{V^{2}}{R}$$

where *V* is the applied voltage and *R* is the resistance of the electrode. It can be seen that the power is proportional to the square of the applied voltage, *V*${}^{2}$. Therefore, similar to electrostatic actuation, by applying an alternating voltage with a frequency, *f*${}_{0}$, across the electrode, mechanical vibration at *f*${}_{0}$ or *f*${}_{0}$/2 can be induced in the structure [26,27]. Power consumption is usually considered one of the main drawbacks of electrothermal actuation. However, power consumption can be reduced by an order of magnitude by removing sources of heat loss from the electrothermal actuators. A study performed on a chevron actuator showed a reduction by an order of magnitude from about 200 mW to about 20 mW after the removal of the substrate (one of the main sources of heat loss) [28].

### 2.3. Piezoelectric Actuation and Read Out

**Figure 3:**
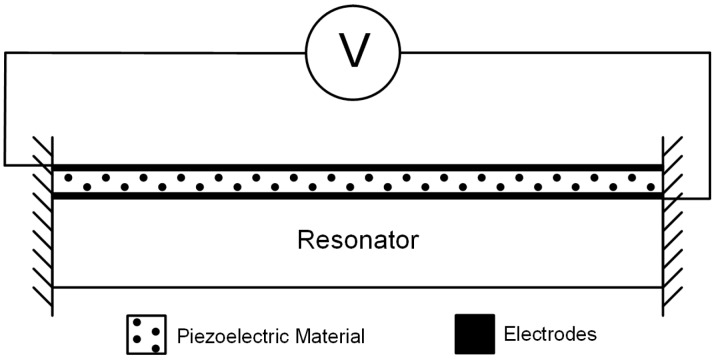
Schematic of piezoelectric transduction on an MEMS resonator.

A piezoelectric material will deform mechanically when an electric field is applied across it, so a piezoelectric layer deposited on top of an MEMS structure can be used for actuation/sensing, provided there is sufficient electro-mechanical coupling between the piezoelectric layer and the MEMS structure. When a voltage is applied across the piezoelectric layer, the resulting electric field will create a strain that will cause a bending moment in the structure, resulting in a vertical deflection. If an alternating voltage is applied to the piezoelectric actuator, as shown in Figure 3, alternating compression and expansion will occur, which results in shear stress that is transformed into a bending moment acting on the MEMS resonator [29]. The structure will be driven into resonance when the applied actuation signal matches the structure’s resonant frequency, *f*${}_{0}$. In addition to inducing mechanical deformation, piezoelectricity is observed in the reverse process, where a material becomes polarised electrically when experiencing mechanical deformation, thus enabling electrical sensing (i.e., read out) of the resonator’s vibration frequency and amplitude. Actuation and read out of the mechanical motion of a structure with piezoelectric materials has been demonstrated to be an excellent transduction technique to be used for MEMS resonators [30].

## 3. Fabrication and Structure Design

Mehregany, Zorman and Cheung have contributed considerably to the advancement of SiC growth [31,32,33,34], material characterisation [35] and device fabrication [16,17,18,31]. Processes, such as atmospheric pressure and low pressure chemical vapour deposition (APCVD and LPCVD), have been shown to be excellent low temperature approaches to grow high quality poly-SiC layers on Si substrates [11,12]. Further contribution to the epitaxial growth of SiC has comes from other research groups [36,37,38,39]. The stress in the SiC layer can affect the curled nature of released structures. The influence on the stress from process parameters during SiC film deposition has been investigated in [38], and techniques for relaxing the stress in released structures have been reported in [34]. SiC can be etched using carbon-based [40], fluorine-based [41] or chlorine-based [42] plasmas. In particular, inductively-coupled plasma reactive ion etching has been demonstrated to be particularly effective (highly selective together with high etch rate) when using fluorine and oxygen chemistries [43]. SiC MEMS have been fabricated by etching and releasing using different separated steps combining dry and wet chemistries [10]. However, when the 3C-SiC layer is grown on a Si substrate or poly-Si sacrificial layer, the structures can be dry etched and released using a one-step process, thus simplifying the device manufacturing greatly [44].

### 3.1. Fabrication Process

The one-step dry etch and release process is particularly convenient for fabricating vertical resonators, such as beams or circular structures to be actuated electrostatically, electrothermally or piezoelectrically [45,46].

**Figure 4:**
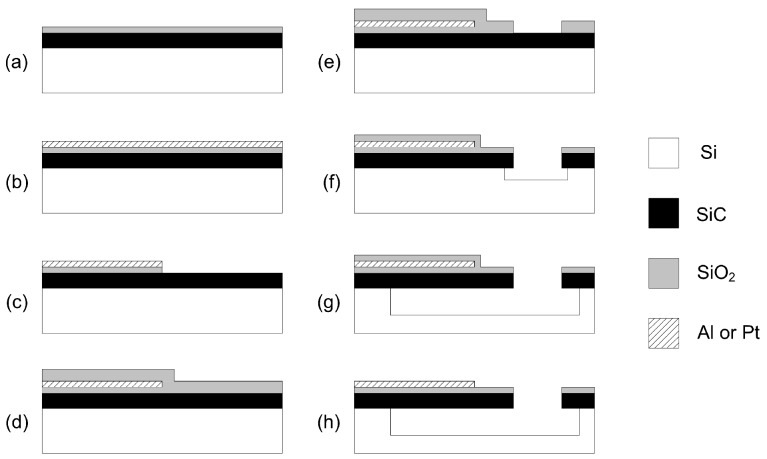
Schematic of the fabrication process for electrothermally-actuated 3C-SiC resonators [27]. ^©^ 2012 IEEE. Reprinted, with permission, from Mastropaolo et al. J. Microelectromech. Syst. 21, 811–821.

Our typical fabrication process used for the fabrication of electrostatically- and electrothermally- actuated vertical 3C-SiC resonators is shown in Figure 4.

The process starts with a 2-μm layer of single crystalline 3C-SiC heteroepitaxially grown on a 100-mm wafer of (100) silicon (Si). The 3C-SiC can be grown utilising a two-step carbonisation-based APCVD process, which has been described in detail elsewhere [47]. Afterwards, a 100-nm layer of oxide is grown thermally (Figure 4a). Then, a metal layer of either aluminium (Al), platinum (Pt) or nichrome (NiCr) is deposited (Figure 4b), patterned photolithographically and etched into the desired shape of electrode using reactive ion etching (RIE) (Figure 4c). After, a 3 μm-thick film of silicon dioxide (SiO${}_{2}$) is deposited on top of the SiC layer (Figure 4d) and patterned photolithographically. The exposed SiO${}_{2}$ is etched away with CHF${}_{4}$/H${}_{2}$ plasma, leaving the oxide mask defining the resonator shape (Figure 4e). The one-step dry etch and release process uses inductively-coupled plasma (ICP) with a mixture of SF${}_{6}$ and O${}_{2}$ and, thus, allowing for etching the SiC layer and releasing partially the Si underneath (Figure 4f) [44] (Figure 2f). Then, the release of the sacrificial Si is adjusted using XeF${}_{2}$ vapour (Figure 4g), and any remaining masking oxide is removed using RIE (Figure 4h).

**Figure 5:**
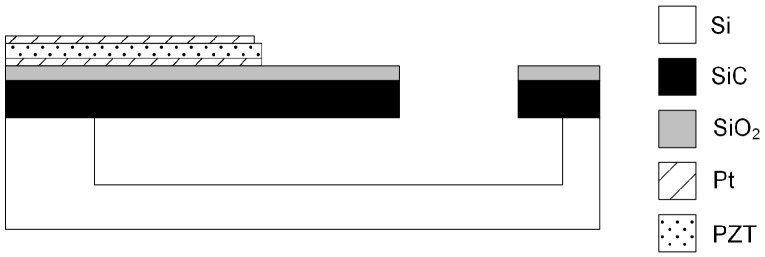
Schematic of piezoelectrically-actuated 3C-SiC resonator.

For resonators utilising piezoelectric transduction, the fabrication process is modified in order to incorporate the piezoelectric ports consisting of a polarised layer of lead zirconate titanate (PZT) sandwiched between layers of Pt. The multilayer Pt/PZT/Pt stack is deposited with a layer thicknesses of 100/500/100 nm, respectively. The Pt layers on top and below the PZT are used as contacts to the PZT, allowing for an actuation voltage to be applied across the PZT layer or for electrical read out of the voltage generated across the PZT layer. The multilayer stack is defined photolithographically; the Pt is dry etched with an argon ion beam tool (acceleration voltage of 250 V and a beam voltage of 500 V); and the PZT is wet etched in a solution of hydrofluoric (HF) and hydrochloric (HCl) acid at a rate of 13 nm/s [48]. The schematic of a SiC resonator with a Pt/PZT/Pt piezoelectric port is shown in Figure 5.

### 3.2. Theory — Design, Dimensions and Frequency

The structural dimensions and the mechanical properties of the material play a critical role when defining the resonant frequency, which is a crucial parameter for timing and telecommunication applications. In addition, different structures, as well as the design and positioning of actuation and sensing electrodes will influence the device’s resonant frequency.

The following formulae describe the fundamental mechanical resonant frequencies (first vertical mode) for cantilevers *f*${}_{C}$, bridges *f*${}_{B}$ and disks *f*${}_{D}$:

(4) $$f_{C}=0.162\sqrt{\frac{E}{\rho }}\frac{t}{L^{2}}$$

(5) $$f_{B}=1.03\sqrt{\frac{E}{\rho }}\frac{t}{L^{2}}$$

(6) $$f_{D}=1.65\sqrt{\frac{E}{\rho }}\frac{t}{D^{2}}$$

where *E* and ρ are the Young’s modulus and the mass density of the resonator material, respectively, *t* is the thickness of the structure, *L* is the length of the cantilever or bridge and *D* is the diameter of the disk [46,49]. It should be noted that Equation (6) is the expression for a disk and only approximates the resonant frequency of a ring with a centre hole (which is included in the design to allow for vapour etching of the Si sacrificial layer). The equations show that, for similar dimensions (i.e., *L* = *D*), the resonant frequency of a disk is approximately 6.4-times higher than a bridge and approximately 10-times higher than a cantilever.

**Figure 6:**
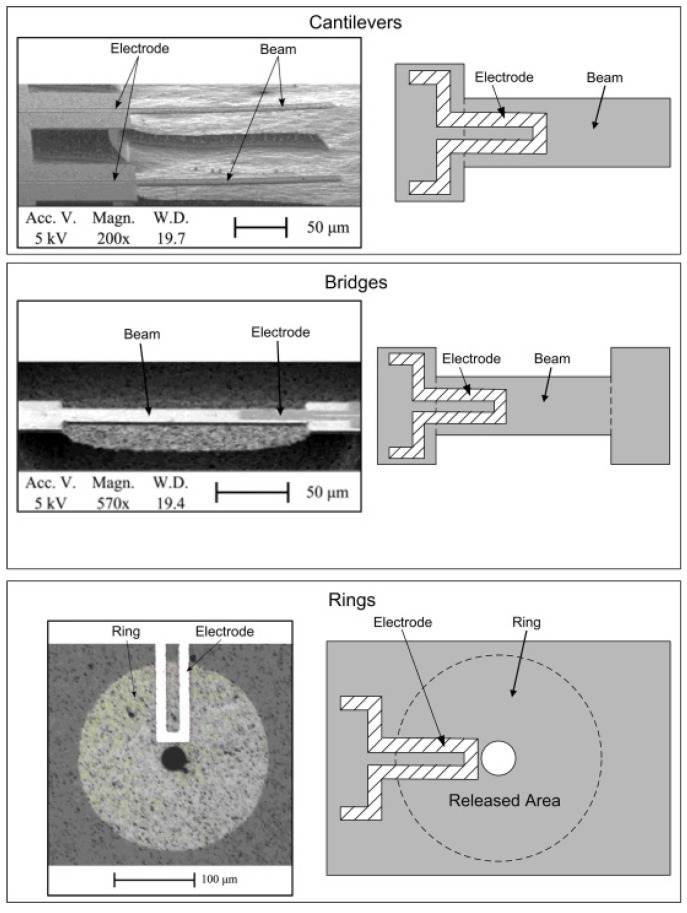
Scanning electron microscopy (SEM) images of electrothermally-actuated SiC cantilevers, bridges and rings along with schematics [27]. ^©^ 2012 IEEE. Reprinted, with permission, from Mastropaolo et al. J. Microelectromech. Syst. 21, 811–821.

Figure 6 shows three examples of the designs of SiC MEMS resonators: single clamped beams (i.e., cantilevers), doubly-clamped beams (i.e., bridges) and disks with a hole in the middle and clamped all-around (i.e., rings) [27]. Previously-reported studies have characterised SiC resonators with lengths and diameters varying between 50 μm and 300 μm.

### 3.3. Optical Measurements

**Figure 7:**
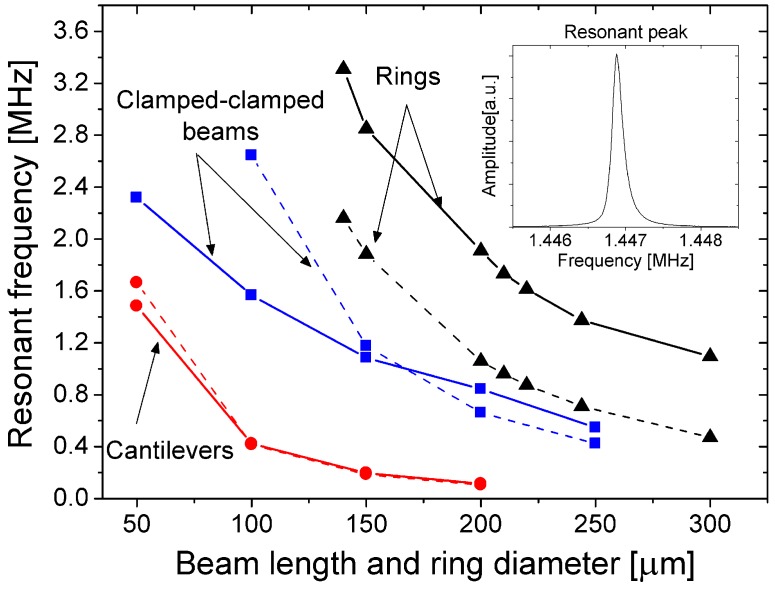
Measured (solid) and theoretical (dashed) dependence of resonant frequency on the structural dimension [27]. ^©^ 2012 IEEE. Reprinted, with permission, from Mastropaolo et al. J. Microelectromech. Syst. 21, 811–821.

The validity of the theoretical Equations (4) to (6) has been explored with resonance measurements on fabricated SiC resonators [27], with the results shown in Figure 7 along with theoretical predictions. The tested devices have been actuated mechanically and their resonant frequencies extracted using a Polytec MSA-400 laser Doppler vibrometer (LDV). The mechanical actuation of the resonators has been achieved by mounting the devices on a piezo disk, which can be set to vibrate at a desired frequency. The mechanical vibration of the piezo disk has been swept through a frequency range around the expected resonant frequency of the resonator, and the mechanical vibrations of the piezo disk have been transferred to the attached MEMS device. As the frequency is being swept, the LDV focuses a laser on the resonator and determines the vibration amplitude by measuring the Doppler shift of the reflected signal with respect to the incident signal [50]. From the amplitude measurements, the resonant frequency and the *Q*-factor of the resonator can be determined.

From Figure 7, in agreement with Equations (4) to (6), a ring resonator possesses a higher resonant frequency than a bridge of similar dimensions, and the measurements confirm that, for the same dimensions, cantilevers possess the lowest resonant frequency. The difference seen between the theory and the measurement data can be explained by noting that the removal of the sacrificial Si layer below the 3C-SiC layer leaves an undercut of up to 30 μm at the anchors, resulting in a longer effective length that lowers the resonant frequency. The influence of the undercut is more dominant for shorter lengths of cantilevers and bridges. For the ring resonators, it can be seen that the measured frequencies are higher than the theoretical predicted values, which could be explained by the fact that Equation (6) describes a disk and does not include the influence of the central hole.

In addition to the device dimensions, the resonant frequency is dependent on the internal stress of the resonator. A previous study [44] with optical measurement of mechanically-excited resonators has shown that bridges, with lengths up to 250 μm, patterned and etched from a 3C-SiC layer grown on a Si substrate, are under tensile stress of 400 to 500 MPa, which increases the resonant frequency relative to the value predicted by Equation (5). Furthermore, the degree to which the fixed anchors restrict the motion of the released portion of the resonator will contribute to the stress, with additional tensile stress increasing the resonance and compressive stress decreasing the resonance.

For ring resonators, the dependence of the resonant frequency on the ring radius has been shown in another study [46], which also featured FEM simulations that determined the effect of internal stress. The simulations modelled an internal stress of 500 MPa for devices with ring diameters up to 400 μm, and the simulated resonant frequencies showed good agreement with the measured results.

## 4. Actuation Methods (with Optical Measurements)

As discussed in Section 2 and Section 3, there are several methods for actuating SiC resonators with different designs and dimensions.

Electrostatic actuation has been used widely in the literature for MEMS devices offering advantages, such as very small actuation currents (intrinsic low power consumption) and fast response. In addition, electrostatic transduction is particularly suitable for driving lateral vibrating structures (such as comb-drives) and for the implementation of the read out. However, electrostatic transduction presents certain drawbacks, including relatively complex fabrication solutions to define the sub-micrometric gaps between the moving structures and the fixed actuation/sensing electrodes. In addition, electrostatically-transduced devices often exhibit relatively high motional impedances and large operating voltages. Electrothermal and piezoelectric actuation offer advantages of combining simpler fabrication processes, relatively low power consumption and low motional impedances, thus representing excellent viable alternatives to electrostatic transduction. However, electrothermal transduction is not particularly suitable when power consumption is critical or as a sensing technique. In these cases, there is a need to implement strategies to minimise power consumption and to combine electrothermal actuation with other transduction mechanisms to perform read out.

This section will review our earlier work on electrostatic and electrothermal actuation on various structures. In particular, the vibration amplitude dependence on the design and location of the input and output ports will be discussed.

### 4.1. Electrostatic Actuation and Optical Read Out

**Figure 8:**
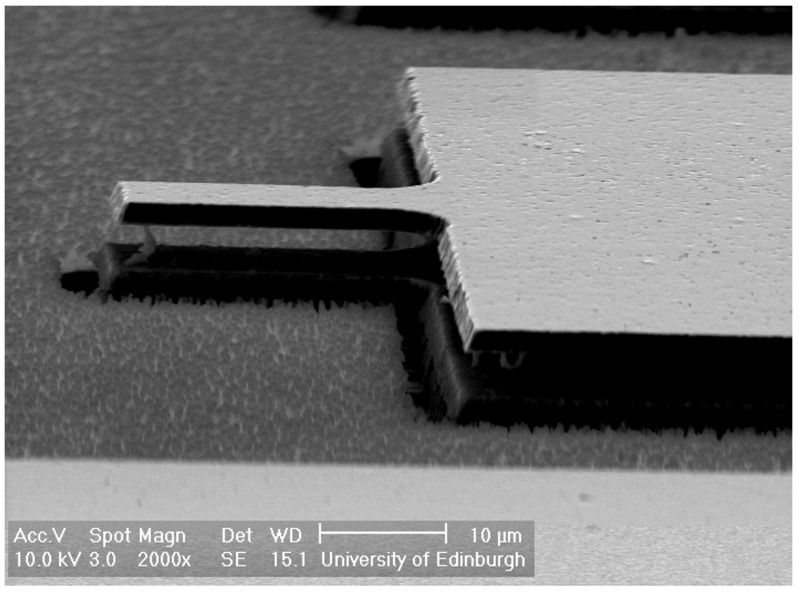
SEM image of the SiC cantilever [45]. Reprinted from Microelecton. Eng., 78–79, Jiang et al., Dry release fabrication and testing of SiC electrostatic cantilever actuators, 78–79, ^©^ 2005, with permission from Elsevier.

An early example of a 3C-SiC resonator is shown in the SEM image in Figure 8: a cantilever has been actuated electrostatically, and the vibration amplitude has been studied as a function of the actuation voltage. In particular, using the fabrication process outlined in Section 3.1, the SiC cantilevers with a length of 25 μm, width of 15 μm, thickness of 2 μm and a 250-nm layer of NiCr deposited on top (covering the entire surface of the cantilever) have been fabricated [45]. This particular type of structure with a relatively simple design and fabrication process can be actuated electrostatically using the NiCr/SiC cantilever as the moveable plate of a capacitor and the Si substrate as the fixed plate.

**Figure 9:**
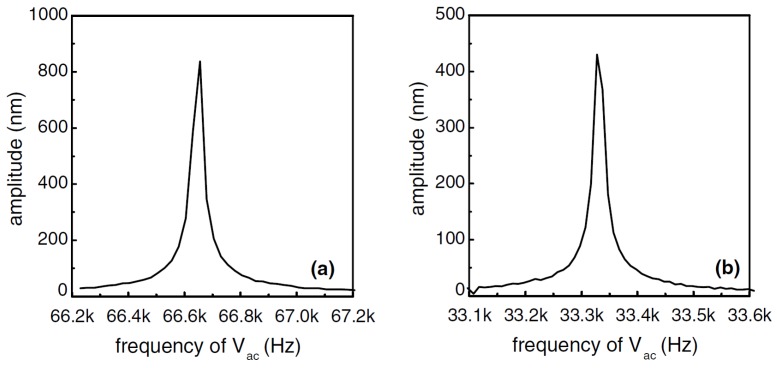
Measured resonant peaks for a 200-μm SiC cantilever with (**a**) *f*${}_{\mathrm{ac}}$ = 66.65 kHz (V${}_{\mathrm{dc}}$ = 0.2 V) and (**b**) *f*${}_{\mathrm{ac}}$ = 33.325 kHz (V${}_{\mathrm{dc}}$ = 0 V) [45]. Reprinted from Microelecton. Eng., 78–79, Jiang et al., Dry release fabrication and testing of SiC electrostatic cantilever actuators, 78–79, ^©^ 2005, with permission from Elsevier.

**Figure 10:**
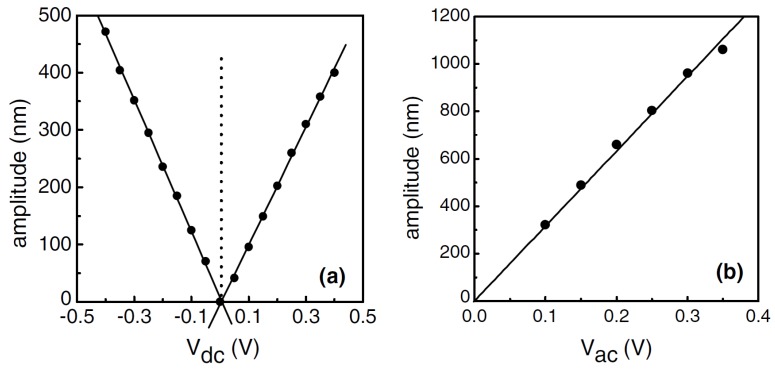
Measured vibration amplitude at resonance as a function of (**a**) DC voltage (V${}_{\mathrm{ac}}$ = 0.3 V) and (**b**) AC voltage (V${}_{\mathrm{dc}}$ = 0.2 V) for 200 μm SiC cantilever [45]. Reprinted from Microelecton. Eng., 78–79, Jiang et al., Dry release fabrication and testing of SiC electrostatic cantilever actuators, 78–79, ^©^ 2005, with permission from Elsevier.

The actuation is achieved by applying a voltage of about 0.5 V between the top (NiCr) and bottom (bulk Si) electrodes. Figure 9 shows the vibration amplitude of the resonator measured optically with LDV as a function of the AC actuation voltage frequency. The resonant peaks can be seen clearly for the applied AC voltage sweeps performed Figure 9a with and Figure 9b without an applied DC voltage, showing that the cantilever has a resonant frequency of 66.65 kHz. The device behaves as expected from Equation (2); with an applied voltage signal with only an AC component driving the structure, the device has a resonant peak at *f*${}_{\mathrm{ac}}$ = *f*${}_{0}$/2 = 33.325 kHz (*V*${}_{\mathrm{dc}}$ = 0 V) [45]. The vibration amplitude at resonance is influenced by the DC and AC voltage components of the applied signal. As expected from Equations (1) and (2), linear relationships between vibration amplitude and applied voltage have been found as shown in Figure 10.

### 4.2. Electrothermal Actuation and Optical Read Out

Cantilever, bridge and ring structures with different electrode designs and materials have been used in the electrothermal actuation of SiC structures. When employing electrothermal actuation, the selection of the electrode design and material plays a key role for maximising the coupling factor and temperature performance of the devices. In the following, the influence of the actuating electrode design on actuation efficiency will be discussed. The most commonly-used electrode materials for electrothermal transduction in the literature are NiCr, Pt or Al, while four different electrode designs have been used: open termination, closed-loop termination (i.e., u-shaped electrodes), full plate covering entire structure (i.e., slab electrode) and interdigitated configuration [26,27,46].

Pt has been shown to be a material that is highly compatible with the high temperature performance of SiC MEMS (TCEs of Pt and SiC are well matched) and SiC resonators actuated with Pt electrodes electrothermally have been demonstrated to achieve higher frequency stability as a function of temperature compared to resonators using Al electrodes [51].

#### 4.2.1. Cantilevers (U-Shaped and Open Termination)

**Figure 11:**
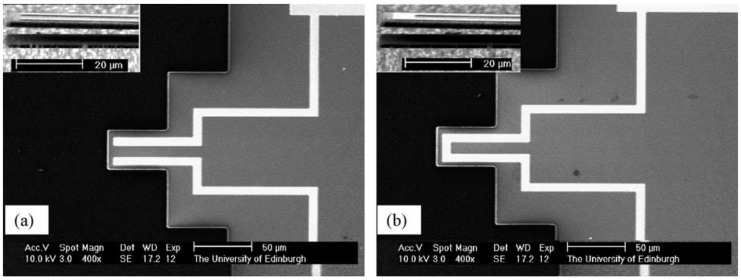
SEM images of 3C-SiC cantilevers with NiCr electrodes for (**a**) open-termination and (**b**) closed termination design [26]. Reprinted from Sens. Act. A: Phys., 128, Jiang et al., SiC cantilever resonators with electrothermal actuation, 376–386, ^©^ 2006, with permission from Elsevier.

Figure 11 shows 3C-SiC cantilevers with open termination and u-shaped electrodes made with a 280 nm-thick NiCr layer actuated electrothermally. Other cantilevers have been fabricated with 500 nm-thick Pt electrodes [26]. The different material and electrode thickness have resulted in a relatively large difference in the resonant frequency for cantilevers with the same length (resonant frequency of NiCr/SiC cantilever 117.12 kHz and Pt/SiC cantilever of 897.4 kHz).

As with electrostatic actuation, it has been found that the vibration amplitude at resonance is related linearly to the value of the DC actuation voltage. In addition, the Pt/SiC device has a higher actuation efficiency (i.e., vibration amplitude at resonance divided by the peak actuation power) than the NiCr/SiC device. The better performance has been attributed to the induced heat being confined to the root of the cantilever for the Pt/SiC device, which occurs because the resistivity ratio of SiC to metal is 2268-times smaller than for the NiCr/SiC device. Additionally, the actuation efficiency of the NiCr/SiC devices is highly dependent on the electrode architecture, with the u-shaped design having 22.5-times larger efficiency compared to open termination.

#### 4.2.2. Bridges (U-Shaped and Slab Electrodes)

**Figure 12:**
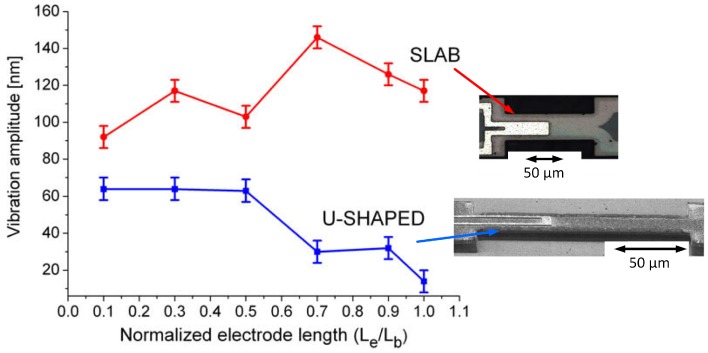
Optically-measured vibration amplitude at resonance as a function of electrode length, *L*${}_{e}$, for an electrothermally-actuated SiC bridge (DC bias voltage 1 V) with length, *L*${}_{b}$ (inset: SEM images of fabricated devices with slab and u-shaped electrodes) [52]. Reprinted from Microelectron. Eng., 87, Mastropaolo et al., Electro-thermal behaviour of Al/SiC clamped-clamped beams, 573–575, ^©^ 2010, with permission from Elsevier.

Further characterisation of the electrothermal actuation mechanism has been performed by comparing the u-shaped and the slab electrode designs (Al electrodes on 3C-SiC bridges) [52]. For the same electrode length, the slab design results in a higher vibration amplitude at resonance than the u-shaped design, a result that finite element method (FEM) simulations have attributed to both a higher maximum temperature at the anchor and average temperature through the structure. For lengths of electrode between 15 μm and 150 μm (i.e., total length of the SiC beam), the amplitude increases as a function of electrode length for the slab design and decreases for the u-shaped design (Figure 12). Measurement and simulation data suggest that the amplitude is influenced and affected by the temperature gradient of the beam and by the amount of overlap area between the Al electrode and the SiC beam. The optimal electrode length for the slab design is 70% of the beam length. A further increase in length results in a lower vibration amplitude, which is probably a consequence of the reaction force of the second anchor damping the increased force arising from the longer electrode. For the u-shaped design, when the optimal electrode length is less than 50% of the beam length, it is believed that bimorph thermal expansion, arising from the different TCEs of Al and SiC, has a greater influence on the actuation force than the thermal expansion of the entire beam, thus resulting in a larger vibration amplitude.

Additional research concerning the electrothermal actuation of SiC bridges with u-shaped Al electrodes has been performed [53]. The findings showed that increasing the electrode width and decreasing the loop spacing can result in larger amplitude at resonance, which has been attributed to a higher temperature through the structure.

#### 4.2.3. Rings (U-Shaped and Interdigitated Electrodes)

**Figure 13:**
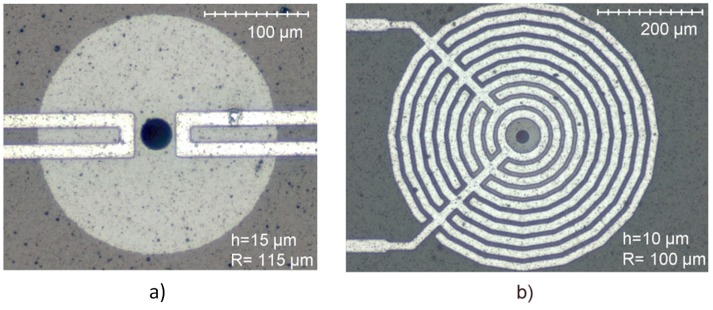
Optical microscope images of Al/SiC ring resonators with: (**a**) a pair of u-shaped electrodes; (**b**) an interdigitated electrode [46]. Reprinted with permission from J. Vac. Sci. Technol. B, 27, Mastropaolo et al., Electrothermal actuation of silicon carbide ring resonators, 3109–3114, ^©^ 2009, American Vacuum Society.

As discussed in Section 3.2, ring structures can achieve higher frequencies, a characteristic that is desirable for RF MEMS. We have investigated the optimisation of the actuation efficiency of SiC ring resonators using different electrode architectures [46]. Al/SiC structures have been fabricated with either one u-shaped electrode, a pair of u-shaped electrodes or an interdigitated electrode (Figure 13). The device with double u-shaped electrodes (Figure 13a) exhibited the highest actuation efficiency. While the single and double u-shaped electrodes induce similar displacement magnitudes, the double configuration demonstrated a more evenly-distributed displacement magnitude, thus potentially enabling easier sensing of deflection and vibration amplitude. The interdigitated design (Figure 13b) is an example of an open-termination electrode, which, similar to earlier studies presented in Section 4.2.1, possesses lower electrothermal actuation efficiency compared to a u-shaped electrode [26]. For the ring resonator devices, the deflection measured when actuated with interdigitated electrodes is 46-times less than the double u-shape electrode, thus exhibiting the same behaviour as for cantilevers in Section 4.2.1.

## 5. Actuation Methods (with Electrical Read Out)

**Figure 14:**
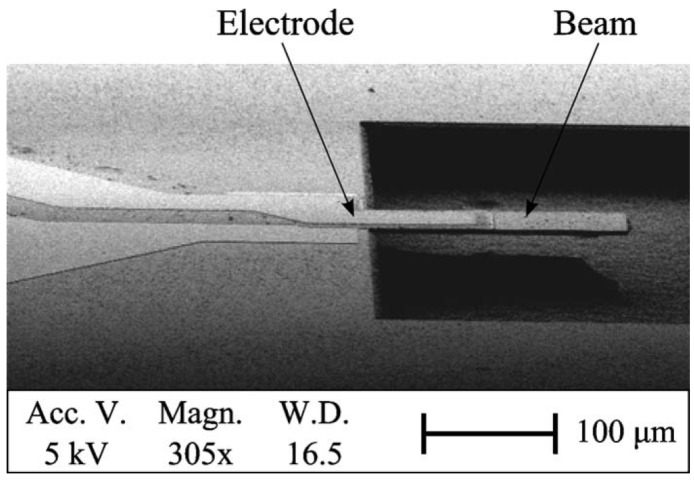
SEM image of a SiC cantilever resonator with a PZT actuating electrode [48]. Reprinted with permission from J. Vac. Sci. Technol. B, 28, Mastropaolo et al., Piezoelectrically driven silicon carbide resonators, ^©^ 2009, American Vacuum Society.

Experimental research work investigating the mechanical behaviour of MEMS resonators often relies primarily on optical testing methods to measure the amplitude and frequency of the structures’ oscillations. While useful at the characterisation stage of the actuation methods and device dimensions, the requirement of using relatively large equipment, such as an LDV, limits its use in practical applications. Bannon et al. provided a major contribution to the development and optimisation of electrical read out of Si resonators focusing in particular on electrostatic transduction used for actuation and sensing [54]. Although electrostatic read out presents similar drawbacks to electrostatic actuation, including the requirement of fabricating sub-micrometric gaps, excellent results were reported showing successful sensing of vertical and contour-mode resonators [39,55]. DeVoe et al. and Piazza et al. focused their work on piezoelectric actuation and read out [29,56,57] where piezoelectric materials, such as zinc oxide and aluminium nitride, were used for fabricating both the resonant structures and the transduction ports. The same piezoelectric read out approach can be used on SiC resonators. For instance, Figure 14 shows an SEM image of a 3C-SiC cantilever with a PZT actuator port fabricated on top of the structure using the process shown in Figure 4 and Figure 5 [48]. Actuation has been achieved by applying an alternating voltage of 0.5 V across the stack Pt/PZT/Pt, which forms the piezoelectric actuating port. The fabricated cantilevers had lengths of 200 μm and 150 μm and showed resonant frequencies in the range 95 kHz to 140 kHz, which was measured by monitoring the variation of electric field across the actuation port electrically. The vibration amplitude is larger for the device with longer actuation ports. From FEM simulations, the higher vibration amplitude observed has been shown to be a result of the larger actuation area, thus maximising the mechanical strain induced to the structure.

### 5.1. Electrostatic Actuation and Read Out

Electrostatic actuation and read out have been used by a number of groups. A few examples are given below.

Electrostatic actuation had been used by Roy et al. to drive some of the first 3C-SiC resonators reported in the literature. The devices were designed as lateral resonating structures and manufactured by surface micromachining [16]. The devices resonated in the range of 10–30 kHz showing reliable operation at up to 950 °C and up to atmospheric pressure achieving *Q*-factor s of 100,000 at pressures of 10${}^{-5}$ Torr with frequency drifts <18 ppm/h.

Wiser et al. have used electrostatic read out based on the detection of motional currents on their poly-SiC vertical bridge resonators [17,18]. The detection scheme is the same as the one described by Nguyen in his work [58]. The devices have been fabricated with a combination of lift-off and wet etching processes to form the submicron air gap allowing for electrostatic actuation. The structures have been actuated into resonance showing frequencies in the range of 1.5–4 MHz with actuation voltages of 5 V (electrostatic tuning has been performed using voltages up to 30 V) [18].

Chang and Zorman reported on the performance of lateral resonating SiC structures (tuning fork design with electrostatic comb-drive) utilising the same detection technique described in [18]. The device performance has been evaluated in terms of operating frequency together with nonlinear behaviour at different pressures, temperatures and actuation voltages [19]. The operating frequency of the SiC structures showed a shift with ambient pressure and variation of about −11 ppm/V to 21 ppm/V with DC bias. SiC structures also showed a relatively stable behaviour against temperature variations with temperature coefficients of 22 ppm/°C between 22 and 60 °C, which are comparable to quartz crystals, but higher than in poly-Si resonators (due to the mismatch of TCE) between the SiC layer and the Si substrate.

Electrostatic actuation and read out had been used further to drive and sense 3C-SiC lateral resonating structures implementing an MEMS-based oscillator, which could be actuated into lateral, rocking and vertical resonances with frequencies of 27.1, 30.3 and 24.2 kHz and *Q*-factors of 13,550, 10,300 and 9480, respectively (10 V DC bias and 1 mTorr pressure) [59]. When operating in rocking mode, the device showed an output signal with power of −17 dBm at 10 V DC bias and at 1 mTorr (minimum phase noise of −78 dBc·Hz${}^{-1}$ at a 12-Hz offset frequency from the carrier).

### 5.2. Electrothermal Actuation and Piezoelectric Read Out

**Figure 15:**
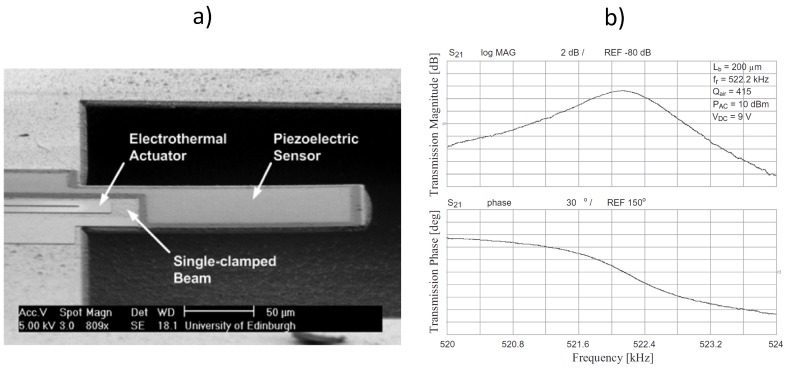
Electrothermally-actuated and piezoelectrically-sensed 3C-SiC cantilever resonator, (**a**) SEM image (**b**) piezoelectrically-measured frequency response, both magnitude and phase (electrothermal actuation with input AC signal power of 10 dBm and DC bias voltage of 9 V) [60]. Reprinted from Microelectron. Eng., 145, Sviličić et al., Tunable MEMS cantilever resonators electrothermally actuated and piezoelectrically sensed, 38–42, ^©^ 2015, with permission from Elsevier.

The combination of electrothermal and piezoelectric transduction represents an interesting approach to be used for actuation and read out, respectively. Such an approach has allowed a cantilever to be driven into vibration electrothermally and its resonant frequency detected electrically without the need of optical measurements. Figure 15a shows an SEM image of the 3C-SiC cantilever fabricated with a Pt electrode for electrothermal actuation and a piezoelectric stack (Pt/PZT/Pt) for read out. As the cantilever vibrates, an alternating voltage is produced across the PZT layer that is at the same frequency as the mechanical oscillations and with an amplitude that is proportional to the mechanical oscillations [60].

A DC bias of 9 V has been applied to the electrothermal electrode along with an AC actuation signal power of 10 dBm. The frequency response of the device has been measured from the output port (Figure 15b), showing a resonant peak at 522 kHz with a *Q*-factor of 415 (measurement performed in air).

**Figure 16:**
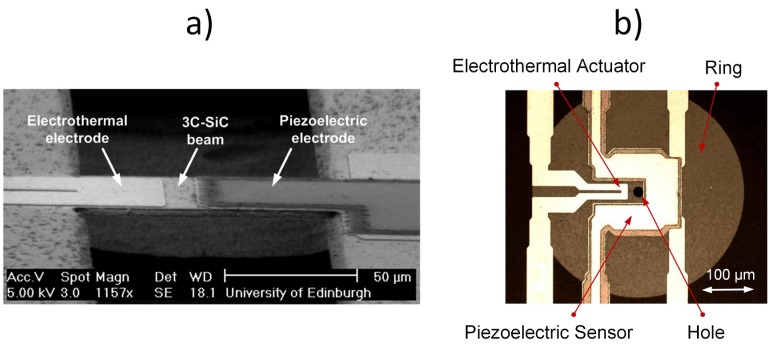
3C-SiC (**a**) bridge [62] and (**b**) ring [63] resonators with a Pt electrode for electrothermal actuation and a piezoelectric port (Pt/PZT/Pt) for read out. ^©^ 2012 IEEE. Reprinted, with permission, from Sviličić et al. Electron Dev. Lett., 33, 278–280 and reprinted from Procedia Eng., 87, Sviličić et al., A MEMS Filter Based on Ring Resonator with Electrothermal Actuation and Piezoelectric Sensing, 1406–1409, ^©^ 2014, with permission from Elsevier.

The combination of electrothermal actuation and piezoelectric read out has been explored in other work using bridges [61,62] and rings [63,64] (Figure 16).

### 5.3. Piezoelectric Actuation and Read Out

Certainly both actuation and read out can be implemented using piezoelectric transduction as reported by DeVoe for vertical bridge resonators made of zinc oxide [29]. In this case, most common structures in the literature show two piezoelectric ports, one for actuation and the other one for sensing the structure’s vibration. 3C-SiC resonators actuated and sensed piezoelectrically have been reported in diverse studies where cantilever, bridge and ring designs have been explored and characterised together with the influence of electrode design on actuation/sensing effectiveness [62,65,66].

**Figure 17:**
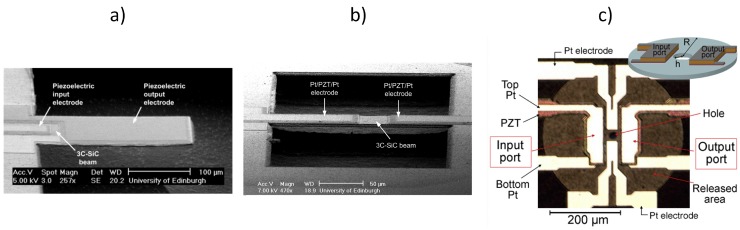
SEM (**a**,**b**) and optical (**c**) images of 3C-SiC structures with ports for piezoelectric actuation and read out: cantilever [65], bridge [66] and ring [67]. Reprinted with permission from J. Vac. Sci. Technol. B, 30, Sviličić et al., Piezoelectrically transduced silicon carbide MEMS double-clamped beam resonators, ^©^ 2012, American Vacuum Society, and reprinted from Microelectron. Eng., 97, Mastropaolo et al., Piezo-electrically actuated and sensed silicon carbide ring resonators, 220–222, ^©^ 2012, with permission from Elsevier.

Figure 17 shows SEM and optical images of 3C-SiC cantilever (Figure 17a), bridge (Figure 17b) and ring (Figure 17c) vertical resonators with integrated piezoelectric ports for actuation and read out. In fact, the cantilever and bridge have two piezoelectric ports, one dedicated for actuation (input port) and the other one for read out (output port). The ring has been designed with the two piezoelectric ports and two additional Pt electrodes, which could be used for electrothermal tuning. The devices have been fabricated with the process shown in Figure 4 and Figure 5. As with electrothermally-actuated devices in Section 5.2, the resonant behaviour of the piezoelectrically-actuated devices has been detected through the voltage output from the piezoelectric output port (labelled in Figure 17c).

In Figure 17a, the input port has been placed at the root of the cantilever leaving a larger area for the output port, thus facilitating the read out of the vibration amplitude. The device resonates at 371 kHz with a *Q*-factor of 385 at atmospheric pressure [65]. In Figure 17b, the piezoelectrically-actuated and -sensed 3C-SiC bridge had been used to compare the resonant behaviour of the device for two different lengths of actuation input ports (i.e., half of the beam length and a third of the beam length). The device with a length of 200 μm resonates at approximately 1 MHz. The bridge that uses the longer input port for actuation results in a larger measured output signal, indicating the existence of higher vibration amplitude [66]. In Figure 17c, the rings showed resonant peaks in the frequency range of 600 kHz to 3.7 MHz for a radius between 65 μm and 200 μm [67]. For these devices, the PZT layer covers a relative large area of the ring, and FEM simulations showed that the rings are under tensile stress and that the stress is dominated mainly by the contribution from the SiC film under the PZT layer. The devices showed a good power handling capacity and linearity (input driving power increased up to 20 dBm with frequency shifts smaller than 100 ppm), and no distortion or hysteresis of the transmission frequency response was observed.

## 6. Frequency Tuning

The possibility of adjusting the resonant frequency of an MEMS resonator is critical in order to compensate for fabrication variations and drifts in frequency occurring over time or as a result of environmental influences. The tuning of the frequency of the device can be performed by varying the input voltage applied to the transduction ports. In this way, spring softening or hardening effects can be induced, and thus, the resonant frequency can be shifted downwards or upwards, respectively. Electrostatic tuning was demonstrated on Si resonators [58] and on SiC devices [18,19], showing that an increase of input voltage induces spring softening effects. Early measurements on 3C-SiC bridge resonators showed that electrostatic tuning can induce a shift from 3.9 MHz down to 2.6 MHz for voltage variation from 5 V to 30 V [18]. Other studies performed on lateral resonators with tuning fork designs and a comb-drive showed shifts of about −11 ppm/V to −21 ppm/V (for input voltage ranging from 20 V to 40 V; above 40 V, a nonlinear behaviour had been observed) [19].

Electrostatic tuning induces only spring softening and thus only allows for downwards shift of the resonant frequency. In addition, electrostatic tuning carries the same drawbacks of utilising electrostatic transduction for actuation and read out.

Therefore, research focused on exploiting electrothermal and piezoelectric transduction for varying the resonant frequency of micro- and nano-structures. The combination of both mechanisms can enable the shift of the resonant frequency of a MEMS resonator either downwards or upwards [27,46,48,52,53,60,61,62,64,66,67]. In particular, electrothermal transduction constitutes an effective solution for frequency tuning while other transduction schemes can be reserved exclusively for actuation and read out. A similar approach was used by Piazza et al. in [57] for zinc oxide resonators where piezoelectric transduction was used for driving and read out and electrostatic transduction used for frequency tuning. Electrothermal actuation and tuning have been demonstrated and investigated on vertical resonators by different research groups [23,26,53]. In our previous work, we have presented a range of vertical resonators and investigated different designs to optimise and combine electrothermal and piezoelectric transduction to be used for actuation, read out and tuning (downwards and upwards) effectively [27,46,48,52,53,60,61,62,64,66,67]. This section will review the current state of research using electrothermal and piezoelectric transduction for tuning the frequency of 3C-SiC MEMS resonators.

### 6.1. Electrothermal Tuning (U-Shaped and Slab Electrodes)

**Figure 18:**
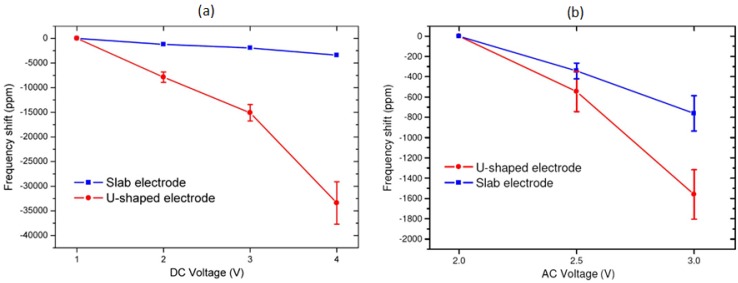
Measured resonant frequency shift of the bridge resonator in response to the change in (**a**) DC or (**b**) AC voltage applied to Pt electrodes for slab and u-shaped designs [27]. ^©^ 2012 IEEE. Reprinted, with permission, from Mastropaolo et al. J. Microelectromech. Syst. 21, 811–821.

When utilising electrothermal transduction, the tuning of the frequency is achieved by inducing a temperature change in the structure, which causes a change in the spring constant of the structure and, depending on structure design, produces additional thermal stress, thus leading to a frequency shift. Electrothermal tuning can be performed along with actuation using the same metal electrode and has been shown to shift the frequency downwards (softening effects). It has been demonstrated experimentally that the electrode design influences the tuning performance [27]. In particular, the electrothermal tuning capability in terms of frequency shift as a function of input voltage for the u-shaped and slab Pt electrode designs shown in Figure 12 has been investigated. By increasing the DC voltage from 1 V to 4 V across the electrode, the initial resonant frequency of 741 kHz has been adjusted down to −35,000 ppm (Figure 18a). By increasing the AC voltage amplitude from 2 V to 3 V, a decrease of the resonant frequency of −1800 ppm has been achieved (Figure 18b). The frequency shift is smaller for AC tuning most likely due to the alternating nature of the signal. Therefore, changing DC voltage or AC voltage could be used for coarse or fine tuning of the resonant frequency, respectively.

**Figure 19:**
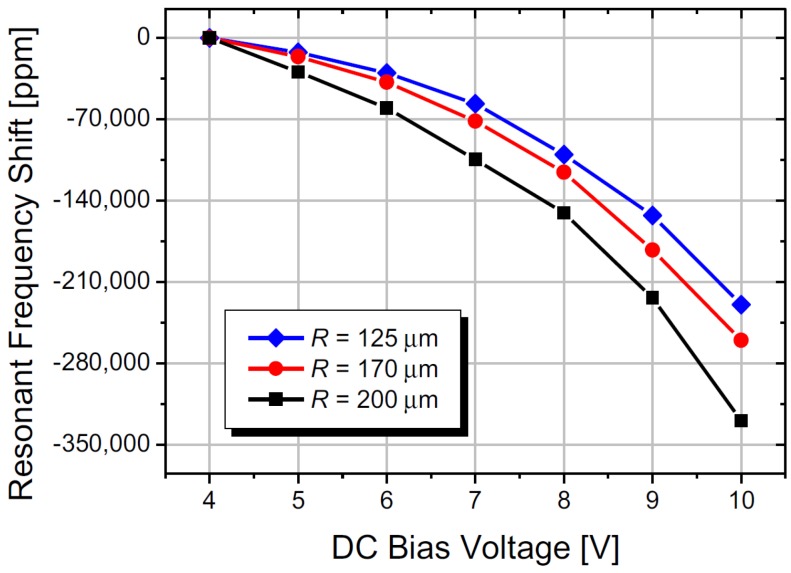
Measured resonant frequency shift of ring resonators in response to the change in DC voltage applied to Pt electrodes [64]. Reprinted from Sens. Act. A: Phys., 226, Sviličić et al., Widely tunable MEMS ring resonator with electrothermal actuation and piezoelectric sensing for filtering applications, 149–153, ^©^ 2015, with permission from Elsevier.

In addition, resonators with u-shaped electrode designs have been observed to be more sensitive to both DC and AC tuning compared to resonators with the slab design. The work presented in [27] had shown that a larger increase in temperature occurs throughout the beam for the u-shaped design as a function of DC bias, rather than just at the anchor for the slab design. Under these conditions, the thermal expansion of the beam is higher and more evenly distributed throughout the beam when using u-shaped electrodes. Therefore, the compressive stress is also larger for u-shaped electrode devices, making them more sensitive to DC bias change, thus achieving a wider tuning range for the same DC bias change compared to the slab electrode design. U-shaped electrodes have been used for tuning cantilever [60], bridge [61] and ring [64] resonators electrothermally, with piezoelectric read out. Cantilever resonators have shown a tuning range of about −1300 ppm (from an initial resonant frequency of 522.6 kHz) for DC bias voltages increasing from 6 V to 11 V [60]. A wider tuning range had been achieved in bridge resonators with a length of 200 μm showing a frequency decrease of about −300,000 ppm when using relatively low magnitudes and small variations of DC input voltages (2 V to 6 V) [61]. Figure 19 shows the response of the resonant frequency to a change in the DC voltage applied to the actuation electrode for ring resonators for three different radii, *R*, with downwards shifts to −350,000 ppm with a DC bias shift of 4 V to 11 V [64].

### 6.2. Piezoelectric Tuning (Small Area vs. Large Area Electrodes)

**Figure 20:**
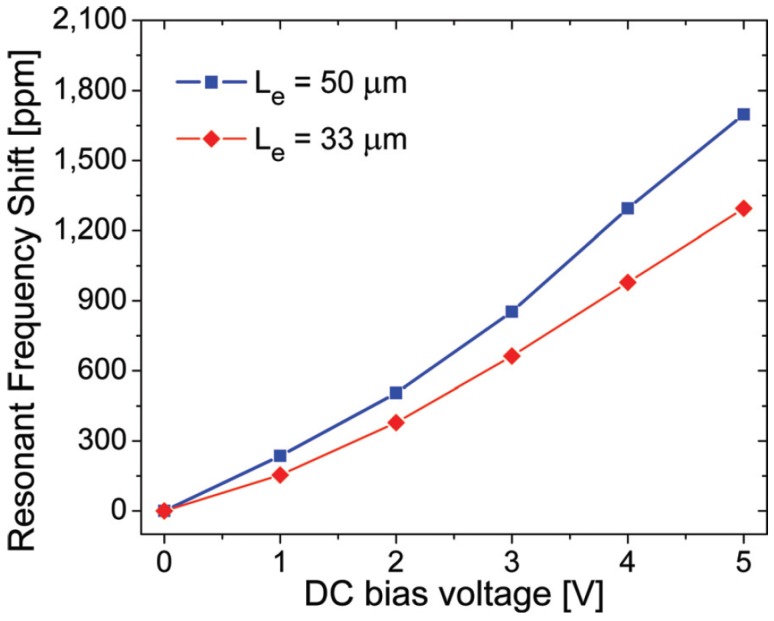
Measured resonant frequency shift of bridge resonators in response to the change in DC voltage applied to a PZT electrode with a length of 33 or 50 μm [66]. Reprinted with permission from J. Vac. Sci. Technol. B, 30, Sviličić et al., Piezoelectrically transduced silicon carbide MEMS double-clamped beam resonators, ^©^ 2012, American Vacuum Society.

Shifting the resonant frequency exclusively downwards could represent a limitation for devices where a variation in the opposite direction (i.e., upwards to higher values) is required. In this case, piezoelectric transduction can be used for tuning the resonant frequency upwards, which, to the authors’ knowledge, was first demonstrated in [66] on SiC devices. The graph depicted in Figure 20 shows the change in resonant frequency as a function of DC bias voltage for a bridge with a length of 200 μm. A frequency increase of up to 1800 ppm has been achieved by increasing the piezoelectric DC input voltage from 0 V to 5 V. Longer input ports exhibited a more sensitive response of the resonant frequency to DC bias due to the increased port area, inducing a greater tensile stress as the DC bias increases (supported by FEM simulations). However, the use of additional DC bias voltages for tuning purposes may lead to nonlinear behaviour, as the increased input power can exceed the maximum handling capabilities of the device.

## 7. Conclusions

There has been a great deal of research in the field of 3C-SiC MEMS resonators in order to exploit the numerous benefits offered from the point of view of performance, adaptability to and reliability for harsh environment operation. The high level of integration, intrinsic to MEMS resonators, makes them very attractive candidates for replacing quartz crystals. MEMS-based oscillators utilising Si as the main structural material, which shows excellent performance, are already commercially available. SiC resonators could enter the MEMS-based oscillator market potentially as an alternative to Si resonators and to cover the portion of the market reserved for harsh environment operation and where high reliability is required.

Since the early years of SiC synthesis, great progress has been made in material characterisation, device fabrication and performance characterisation. 3C-SiC can be synthesised epitaxially on Si substrates using APCVD or LPCVD, thus largely reducing manufacturing costs. In addition, 3C-SiC has outstanding mechanical properties that are comparable to the other SiC polytypes. For these reasons, most of the work reported has used 3C-SiC for developing a variety of resonating structures. Electrostatic, electrothermal and piezoelectric actuation methods have been employed and investigated for developing SiC MEMS oscillators, mixers and filters. The main body of literature has followed the progress made for Si devices and investigated further on the performance of 3C-SiC devices in terms of actuation effectiveness and the reliability of devices at different atmospheric conditions.

3C-SiC vertical and lateral resonators have been reported in the literature for the last 20 years. Our work used a one-step etching and release method using fluorine-based plasmas for manufacturing vertical resonators, but could also be employed for making lateral resonators. Vertical beam and ring resonators with characteristic dimensions between 25 μm and 300 μm have shown operation in the frequency range of 20 kHz to 3.5 MHz with quality factors up to 10,000 in a vacuum. Such results prove that 3C-SiC devices can be utilised for implementing reference clock oscillators. Rings should be used when high frequency operation is required.

Electrostatic actuation has been preferred in certain cases, in particular on lateral resonators, and has been shown to be an excellent driving and sensing method with good tuning effectiveness, but presenting drawbacks, such as complex fabrication to create sub-micrometric gaps and relatively large driving voltages. A 3C-SiC lateral resonator driven and sensed electrostatically (folded-beam design with comb-drive and read out) has been used successfully to implement an MEMS-based oscillator achieving a quality factor of 10,300 at 30.2 kHz for a rocking mode resonance at 1 mTorr.

When aiming to simplify the fabrication process (possibly maximising yield) together with minimising driving voltages, electrothermal and piezoelectric techniques have been chosen as good alternatives to electrostatic actuation for performing transduction on vertical resonators. The effectiveness of electrothermal actuation for driving and frequency tuning and can be maximised by optimising the design of the driving/tuning electrode port. U-shaped electrodes covering up to half of the structure’s characteristic length give a good compromise between vibration amplitude and tuning range. The larger vibration amplitudes achieved by slab electrodes architecture, for a given actuation voltage signal, could lead potentially to nonlinearities in the frequency response. Electrothermal actuation can be combined with piezoelectric transduction for read out and further frequency tuning. The resonators have been actuated with voltages as low as 0.5 V and the resonance detected piezoelectrically. In particular, for the purposes of tuning the resonant frequency, electrothermal transduction allows for lowering the frequency, and piezoelectric transduction allows for raising the frequency. However, it is important to note that the range of frequency variation is higher for electrothermal tuning (−300,000 ppm with a DC bias shift from 2 V to 6 V on 200 μm-long bridge resonators) compared to piezoelectric (+1800 ppm with DC bias shift from 0 V to 5 V on 200 μm-long bridge resonators).

This paper has reviewed and summarised the literature on 3C-SiC resonators. The devices have been shown to be good alternatives to Si for developing MEMS-based oscillators. However, the major drawback affecting 3C-SiC resonators is found in the epitaxial nature of the SiC used. Although 3C-SiC’s mechanical properties are comparable to 4H-SiC or 6H-SiC, problems could arise due to the mismatch with Si substrate (different TCEs), thus affecting the reliability of the device performance. The limitation arising from TCE mismatch between 3C-SiC and Si substrates could be overcome by moving towards the development of resonators made from bulk wafers of hexagonal SiC polytypes. Such a solution would increase the manufacturing cost potentially, but will make SiC resonators unique players in the MEMS-based oscillator market.
